# Laser-Induced In Situ Crystallization of Hybrid Manganese(II) Bromide Arrays for X-Ray Imaging

**DOI:** 10.3390/s26082373

**Published:** 2026-04-12

**Authors:** Zhaoran Lin, Guansheng Xing, Wei Wang, Bing Chen

**Affiliations:** College of Electronic and Optical Engineering and College of Flexible Electronics (Future Technology), Nanjing University of Posts and Telecommunications, Nanjing 210023, China

**Keywords:** metal-halide, femtosecond laser processing, in situ crystallization, pixelated arrays, X-ray imaging

## Abstract

**Highlights:**

**What are the main findings?**

- Laser-induced in situ crystallization can directly convert hybrid manganese(II) bromide glass into crystalline pixel arrays. Starting from glassy (BuTPP)_2_MnBr_4_, we used a femtosecond laser to achieve localized and controllable crystallization, through which the crystalline arrays can be structurally patterned inside the glass matrix rather than fabricated by conventional crystal-growth methods.
- The laser-written pixelated (BuTPP)_2_MnBr_4_ arrays show improved luminescence and enable high-resolution X-ray imaging. Compared with the glassy state, the crystallized pixel arrays exhibit significantly enhanced photoluminescence, and they can be used for X-ray imaging with a spatial resolution of 10 lp mm^−1^.

**What are the implications of the main findings?**

- This work provides a new structural-engineering route for hybrid scintillators. Instead of only tuning chemical composition, it shows that local phase/structure control can be used to improve scintillation performance.
- This work opens a practical pathway toward customizable high-resolution scintillator devices. Because the pixel arrays are formed by direct laser-writing, this strategy could support programmable, integrated, and potentially scalable X-ray imaging devices based on hybrid metal-halide.

**Abstract:**

Hybrid metal-halide scintillators are promising for X-ray imaging, but direct fabrication of patterned arrays with high spatial precision remains challenging. Here, we report a laser-induced in situ crystallization strategy for constructing pixelated scintillator arrays from a melt-processable manganese(II) bromide glass precursor, (BuTPP)_2_MnBr_4_ (BuTPP^+^, butyltriphenylphosphonium). The (BuTPP)_2_MnBr_4_ undergoes low-temperature glass formation and can be selectively recrystallized under femtosecond laser irradiation, enabling programmable spatial patterning. Structural analyses confirm the recovery of the crystalline phase after laser writing, while photophysical measurements show markedly enhanced photoluminescence and radioluminescence compared with the glassy state. Benefiting from efficient X-ray-to-light conversion and precise array definition, the patterned scintillators exhibit a high light yield of 24,600 photons MeV^−1^, an X-ray detection limit of 4.89 µGy_air_ s^−1^, and a spatial resolution of 10 lp mm^−1^. This work establishes the laser-induced in situ crystallization strategy as an effective route to integrated hybrid scintillator arrays and offers a versatile platform for customizable and low-temperature processed X-ray imaging devices for imaging uses.

## 1. Introduction

Scintillator-based X-ray detection has become indispensable in medical diagnostics, security inspection, industrial nondestructive testing, and high-energy radiation monitoring [1,2,3]. By converting high-energy X-ray photons into detectable visible-light signals, scintillators serve as the core functional materials in indirect X-ray imaging. To date, commercial scintillators are dominated by all-inorganic compounds such as cerium-doped Lu_3_Al_5_O_12_ (LuAG:Ce), Bi_4_Ge_3_O_12_ (BGO), and thallium-activated CsI (CsI:TI), because of their high sensitivity and mature device integration [4,5]. However, all-inorganic scintillators are often associated with high processing temperatures, energy-intensive crystal growth, limited shape adaptability, and relatively high manufacturing cost, which can restrict customized device fabrication [6]. In this regard, organic–inorganic hybrid metal-halides (OIMHs) have emerged as a promising new family of scintillators owing to the low-temperature processability and potentially lower fabrication cost [7,8,9,10]. More importantly, the structural diversity and compositional tunability of OIMHs provide a versatile platform for regulating coordination environment, exciton behavior, and emission characteristics, making them highly attractive for high-performance scintillation.

Currently, OIMHs used in X-ray imaging are predominantly available in the form of single crystals [11], thin films [12], and glass [13]. Single crystals typically exhibit high luminescence efficiency and well-defined structures, but their growth is often time-consuming, size-limited, and difficult to spatially control [14]. Thin films are more compatible with scalable deposition, yet they commonly suffer from limited thickness, reduced transparency, and substantial light scattering arising from grain boundaries or surface roughness, which are unfavorable for high-quality scintillation output [15]. In contrast, glassy OIMHs offer several unique advantages, including facile melt processing, good optical uniformity, large-area fabrication capability, and compatibility with diverse substrates and device geometries [16]. Nevertheless, the glassy state usually compromises the local structural order of luminescent centers, resulting in reduced photoluminescence quantum yield and weaker scintillation performance compared with their crystalline counterparts. Therefore, although considerable effort has been devoted to the composition engineering of hybrid metal-halide scintillators [17,18,19], strategies that can simultaneously preserve the processing advantages of glasses while locally recovering the superior optical properties of crystals remain highly desirable. This challenge points to the importance of structural engineering, which remains comparatively underexplored in OIMH scintillators.

Among structural engineering approaches, pixel-array formation is particularly attractive for X-ray imaging [20,21]. Pixelated scintillator architectures can confine optical signals within discrete units, suppress lateral photon spreading, and thereby reduce optical crosstalk between neighboring regions [22,23,24]. These features are critical for improving modulation transfer, edge fidelity, and spatial resolution in scintillation imaging [25]. Conventionally, scintillator pixel arrays are fabricated through mold-assisted growth, dicing, and templating [26,27,28]. While effective, these methods often involve multiple fabrication steps, limited spatial flexibility, or poor compatibility with fragile and process-sensitive hybrid materials. For OIMHs in particular, direct construction of high-resolution, well-defined pixel arrays remains challenging because they are generally sensitive to heat, solvents, and mechanical processing. Accordingly, a fabrication route that enables direct, localized, and programmable patterning inside a bulk hybrid matrix would be highly advantageous for integrating OIMH scintillators into high-resolution imaging devices.

Laser-induced in situ crystallization offers such a possibility. Ultrafast lasers, especially femtosecond lasers, are powerful tools for three-dimensional micro-/nano-structuring of transparent media because they can deposit energy with high spatial precision and induce localized modification without globally damaging the surrounding material [29,30]. Femtosecond-laser irradiation can also trigger local heating, phase transformation, and site-selective crystallization inside glassy matrices [31]. This capability is particularly appealing for hybrid metal-halide glasses, where one may envision starting from a processable amorphous precursor and then using focused laser irradiation to selectively reconstruct emissive crystalline domains at desired locations. Such a strategy would combine the manufacturing advantages of glass with the superior luminescence of the crystalline phase, while simultaneously enabling direct laser-writing of scintillating pixel arrays. Therefore, rather than relying solely on chemical composition screening or ex situ assembly, laser-induced in situ crystallization provides a new route to structurally program scintillators with both spatial definition and enhanced optical functionality.

Herein, we demonstrate the concept of laser-induced in situ crystallization using (BuTPP)_2_MnBr_4_ (BuTPP^+^ = butyltriphenylphosphonium) as a model hybrid manganese(II) bromide system. A transparent glassy precursor is first prepared by melt quenching, and localized recrystallization is then induced by femtosecond-laser irradiation through controlled photothermal processing. This laser-written phase transition reconstructs ordered Mn–Br coordination environments within selected regions and gives rise to pixelated crystalline arrays with markedly enhanced luminescence. Benefiting from the combination of glass processability, laser programmability, and crystal-like scintillation performance, the resulting crystalline (BuTPP)_2_MnBr_4_ arrays enable high-resolution X-ray imaging with a spatial resolution of 10 lp mm^−1^. This work establishes laser-induced in situ crystallization as an effective structural-engineering strategy for hybrid metal-halide scintillators and offers a new pathway toward customizable and integrated X-ray imaging devices.

## 2. Materials and Methods

### 2.1. Materials

Butyltriphenylphosphonium bromide (C_22_H_24_PBr, abbreviated as BuTPP-Br, 97%), manganese bromide tetrahydrate (MnBr_2_·4H_2_O, 98%), methanol (CH_3_OH, 99.7%), and dichloromethane (CH_2_Cl_2_, 99.5%) were purchased from Aladdin Biochemical Technology Co., Ltd. (Shanghai, China). All chemicals were used as received without further purification.

### 2.2. Synthesis of (BuTPP)_2_MnBr_4_ Single Crystals

(BuTPP)_2_MnBr_4_ single crystals were synthesized by a solvent-evaporation method. In a typical procedure, MnBr_2_·4H_2_O (5 mmol) and BuTPP-Br (10 mmol) were weighed and dissolved in a mixed solvent of methanol (10 mL) and dichloromethane (10 mL). The resulting solution was stirred at room temperature until it became clear and was then left in a fume hood at room temperature (~25 °C) to allow slow solvent evaporation and crystal growth.

### 2.3. Synthesis of (BuTPP)_2_MnBr_4_ Glass Through Melt Cooling

The (BuTPP)_2_MnBr_4_ crystals were placed in a quartz crucible and heated to 185 °C until fully molten. The melt was then allowed to cool naturally to room temperature in the crucible to obtain the glassy phase.

### 2.4. Synthesis of (BuTPP)_2_MnBr_4_ Crystalline Pixelated Arrays Through Laser Heating

A 1030 nm femtosecond laser with a repetition rate of 100 kHz and a pulse duration of 300 fs was focused through a 50× objective lens (NA = 0.65). A computer-controlled three-axis translation stage was used to control the sample position and scanning speed, while the laser power was adjusted using an attenuator. The focal (depth of crystallization) was set at 60 μm below the surface of (BuTPP)_2_MnBr_4_ glass. The (BuTPP)_2_MnBr_4_ glass was mounted on the stage and irradiated under programmed conditions. Crystalline pixelated arrays of (BuTPP)_2_MnBr_4_ were generated by software-controlled definition of the dot-matrix size and spacing.

### 2.5. Molecular Electrostatic Potential Calculation

The molecular electrostatic potentials of hybrid manganese(II) bromides with different carbon-chain lengths (1C–6C) in the organic cations were calculated by DMol3 software package from Materials Studio 2020. All calculations employed the Becke–Lee–Yang–Parr (BLYP) exchange-correlation functional within the generalized gradient approximation (GGA) to describe the exchange-correlation interactions between electrons [32,33].

### 2.6. Characterizations

Single-crystal XRD data were collected on a Rigaku XtaLAB Synergy X-ray diffractometer (Rigaku Corporation, Tokyo, Japan) equipped with a Mo Kα X-ray (λ = 0.71073 Å) tube. Powder XRD data were collected on a Bruker D8 Advance A25 diffractometer (Bruker Corporation, Billerica, MA, USA) with Cu Kα X-ray (λ = 1.54056 Å). Thermogravimetry analysis (TGA) was performed on Netzsch TG STA 449F5 Jupiter (NETZSCH, Bavaria, Germany) from 25 to 800 °C using a 10 °C min^−1^ ramping rate under nitrogen gas flow (40 mL min^−1^). Differential scanning calorimetry (DSC) analysis was carried out on Netzsch DSC 200F3 (NETZSCH, Bavaria, Germany) at a heating rate of 10 °C min^−1^ under a nitrogen gas atmosphere. The photoluminescence spectra were measured using an F-4700 fluorescence spectrometer (Hitachi, Tokyo, Japan). The PL lifetime and PLQY measurements were conducted using an FLS980 spectrometer (Edinburgh Instruments, Edinburgh, UK). The transmitted spectrum was measured using a Shimadzu UV-2600 UV-Vis spectrometer (Shimadzu Corporation, Kyoto, Japan). The RL spectrum was collected through an QE PRO fiber optic spectrometer (Ocean Optics, Orlando, FL, USA) coupled with a miniX2 X-ray tube (Amptek Inc., Bedford, MA, USA) as the excitation source. Commercial LuAG:Ce scintillator (Shuojie Crystal Materials, Shanghai, China) with a LY of 22,000 photons MeV^−1^ was used as the reference.

## 3. Results and Discussion

**Figure 1:**
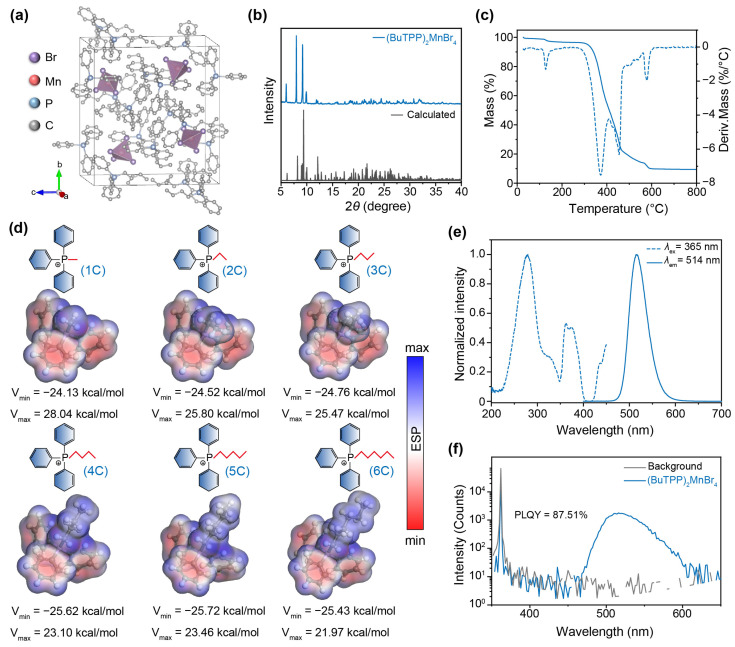
Characterizations of (BuTPP)_2_MnBr_4_ crystals. (**a**) The 3D plots of (BuTPP)_2_MnBr_4_ single crystal structure. (**b**) Powder XRD pattern of (BuTPP)_2_MnBr_4_ crystals. The calculated result is derived from the single-crystal XRD data. (**c**) Thermogravimetric (solid line) and derivative thermogravimetric (dashed line) curves of (BuTPP)_2_MnBr_4_ crystals, respectively. (**d**) Molecules (top panel) and electrostatic potential plots (bottom panel) of hybrid manganese (II) bromides with different carbon-chain lengths (1C–6C) in the organic cations, respectively. (**e**) The excitation (dashed line) and emission (solid line) spectra of (BuTPP)_2_MnBr_4_ crystals, respectively. (**f**) The PLQY plot of (BuTPP)_2_MnBr_4_ crystals under 365 nm excitation.

We used a simple solution-evaporation and room-temperature-crystallization method for the synthesis of (BuTPP)_2_MnBr_4_ single crystals, using MnBr_2_·4H_2_O as the manganese precursor and BuTPP-Br as the organic precursor. As shown in Figure 1a, (BuTPP)_2_MnBr_4_ crystallizes in the monoclinic *P*2_1_*/n* space group with lattice parameters of *a* = 10.8592 Å, *b* = 21.7967 Å, and *c* = 19.6633 Å. The full crystallographic parameters are summarized in Table S1. For the coordination structure of (BuTPP)_2_MnBr_4_, each Mn^2+^ center is tetrahedrally coordinated with four Br^−^ anions to form an isolated [MnBr_4_]^2−^ unit that is separated by bulky BuTPP^+^ organic cations, giving rise to a typical zero-dimensional electronic structure [34,35]. The powder XRD pattern of the bulk (BuTPP)_2_MnBr_4_ crystals agrees well with the simulated pattern and exhibits multiple sharp Bragg peaks, confirming the phase purity and high crystallinity of the as-synthesized sample (Figure 1b).

To evaluate the thermal robustness and explore the thermodynamic property, thermogravimetric analysis (TGA) was performed on the (BuTPP)_2_MnBr_4_ crystals (Figure 1c). The TGA curve demonstrates that (BuTPP)_2_MnBr_4_ crystals remain stable up to ~330 °C, above which pronounced mass change is observed, consistent with decomposition of the organic component and the formation of MnBr_2_ species with a ~69% mass loss. To further understand the role of organic cations in regulating the thermodynamic behavior of hybrid manganous bromides, we compared the electrostatic potentials of phosphonium cations with alkyl chain lengths from 1C to 6C (Figure 1d). Compared with the short-chain analogs (e.g., 1C, 2C, and 3C), the 4C cation (i.e., BuTPP^+^) exhibits a more negative V_min_, indicating higher electron density on its phosphorus atom and stronger electron-donating ability, which facilitates the formation of stronger σ-coordination bonds with metal centers. In contrast, longer alkyl chains (e.g., 5C and 6C) may introduce additional steric hindrance. BuTPP^+^, therefore, appears to provide a favorable balance between interaction strength and steric compatibility, which is beneficial for subsequent melt processing.

In a further set of experiments, the photoluminescence (PL) of (BuTPP)_2_MnBr_4_ crystals was investigated by recording their excitation and emission spectra (Figure 1e). According to the steady-state PL spectrum, the (BuTPP)_2_MnBr_4_ crystals exhibit a broad emission peak centered at 514 nm under 365 nm excitation, assigned to the characteristic ^4^T_1_(G) → ^6^A_1_ (^6^S) transition of Mn^2+^ ions [36]. By monitoring the emission wavelength at 514 nm, the excitation of (BuTPP)_2_MnBr_4_ crystals contains three broad bands at 250–350, 350–400, and beyond 430 nm, corresponding to ligand-to-Mn^2+^ energy transfer, ^6^A_1_(^6^S) → ^4^E/^4^T_2_(^4^D), and ^6^A_1_(^6^S) → ^4^A_1_/^4^T_2_(^4^G) transitions of Mn^2+^ ions, respectively. We also quantitatively evaluated the luminescence efficiencies of (BuTPP)_2_MnBr_4_ crystals by measuring the PLQY (Figure 1f). The crystalline (BuTPP)_2_MnBr_4_ exhibits a high PLQY of 87.51% under 365 nm excitation. Such a high value is ascribed to the large Mn–Mn distance to suppress energy dissipation, which is deemed to cause optical quenching in phosphors [37].

**Figure 2:**
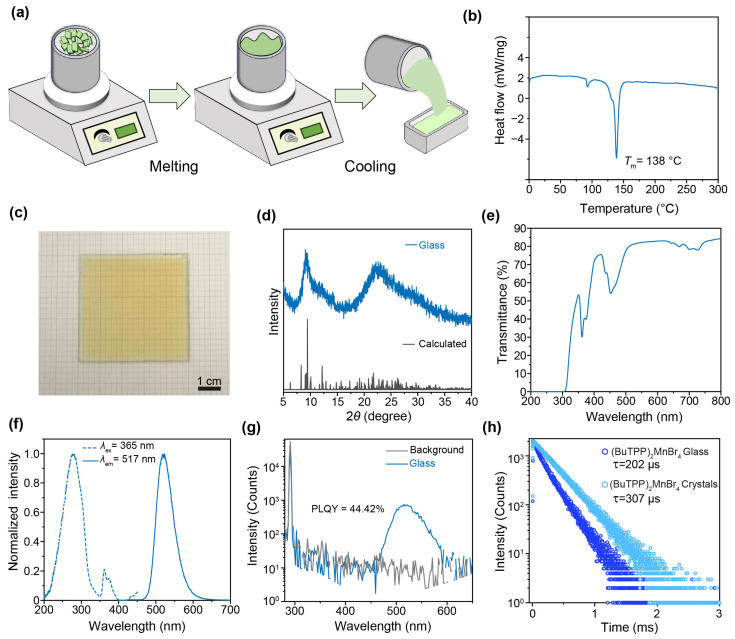
Synthesis and Characterizations of (BuTPP)_2_MnBr_4_ glass. (**a**) Schematic diagram of the preparation of (BuTPP)_2_MnBr_4_ glass. (**b**) DSC curve of (BuTPP)_2_MnBr_4_ crystals. (**c**) A photograph of the as-synthesized (BuTPP)_2_MnBr_4_ glass. (**d**) Powder XRD pattern of (BuTPP)_2_MnBr_4_ glass. (**e**) Transmittance spectrum of the (BuTPP)_2_MnBr_4_ glass. (**f**) The excitation (dashed line) and emission (solid line) spectra of (BuTPP)_2_MnBr_4_ glass, respectively. (**g**) PLQY plot of (BuTPP)_2_MnBr_4_ glass under 291 nm UV excitation. (**h**) A comparison of decay curves for the glassy and crystalline (BuTPP)_2_MnBr_4_, respectively.

We then employed a facile melting-cooling method for the synthesis of (BuTPP)_2_MnBr_4_ glass, as shown in Figure 2a. The differential scanning calorimetry (DSC) curve of (BuTPP)_2_MnBr_4_ crystals shows a melting temperature (*T*_m_) of 138 °C, which is substantially lower than the decomposition temperature and therefore enables melt processing without obvious thermal degradation (Figure 2b). Based on this thermal window, the polycrystalline precursor was heated to 185 °C to form a homogeneous melt and then cooled to room temperature to yield a glassy sample (Figure 2c). Unlike the crystalline counterpart, the (BuTPP)_2_MnBr_4_ glass exhibits broad diffuse features in the powder XRD pattern rather than sharp Bragg peaks, confirming the loss of long-range order (Figure 2d) [38]. In addition, the (BuTPP)_2_MnBr_4_ glass remained chemically stable after being stored at 30% humidity for 7 days, exhibiting a good environmental stability (Figure S1).

We next examined the optical properties of the as-synthesized (BuTPP)_2_MnBr_4_ glass. The glass shows high transmittance in the visible region (>80% over 510–665 nm), as shown in Figure 2e. The transmittance decreases near 360 and 450 nm, consistent with Mn^2+^-related absorption. Similarly to the crystalline (BuTPP)_2_MnBr_4_, the glass exhibits green emission excited by 365 nm UV light, and its emission peak is centered at 517 nm (Figure 2f). The closely matched PL spectra indicate that the tetrahedrally coordinated Mn^2+^ emissive centers are largely preserved. The PLQY value of the glass reaches 44.42%, lower than that of the crystal, which is consistent with additional nonradiative loss in the disordered state (Figure 2g). From the time-resolved PL spectra in Figure 2h, decay time decreases from 307 μs in the (BuTPP)_2_MnBr_4_ crystal to 202 μs in its glass form, indicating enhanced nonradiative relaxation in the amorphous phase [39].

**Figure 3:**
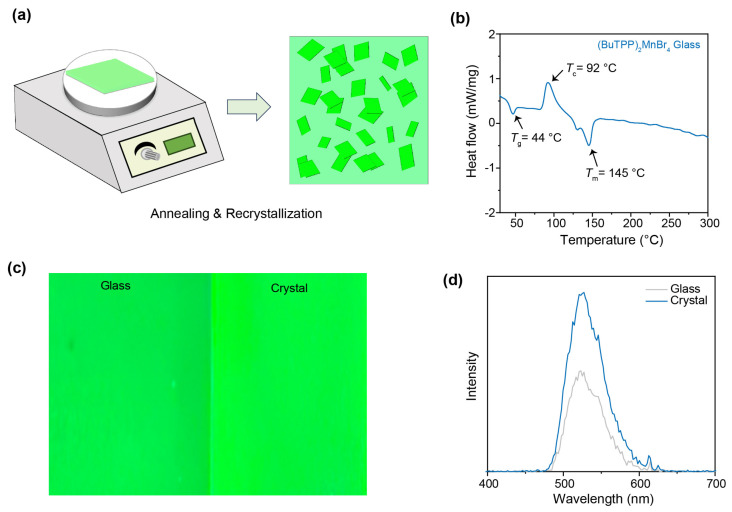
Synthesis and Characterizations of (BuTPP)_2_MnBr_4_ crystals by in situ annealing and crystallization. (**a**) Schematic diagram of the preparation of (BuTPP)_2_MnBr_4_ crystals by annealing and crystallization. (**b**) DSC curve of (BuTPP)_2_MnBr_4_ glass. (**c**) Comparative photographs of the (BuTPP)_2_MnBr_4_ glass and crystals. (**d**) Comparative emission spectra of (BuTPP)_2_MnBr_4_ glass and crystals.

After warming the (BuTPP)_2_MnBr_4_ glass in a heating stage, we noticed that the glass sample would recrystallize into polycrystals (Figure 3a). The DSC curve of the (BuTPP)_2_MnBr_4_ glass reveals a glass transition temperature (*T*_g_) of 44 °C, followed by a distinct exothermic peak at 92 °C, which was attributed to the crystallization temperature (*T*_c_) of the (BuTPP)_2_MnBr_4_ phase (Figure 3b). The subsequent endothermic peak was ascribed to the *T*_m_ of the (BuTPP)_2_MnBr_4_ glass. According to the Turnbull criterion, glass formation is favored when TRG = *T*_g_/*T*_m_ (in Kelvin) exceeds 0.67 [40]. For (BuTPP)_2_MnBr_4_, the TRG value is approximately 0.75, consistent with its good glass-forming ability. Such low-temperature processability is advantageous for fabricating temperature-sensitive photonic or imaging devices, where high-temperature processing can damage substrates or degrade interfaces. Optical microscopy under UV excitation further shows that the recrystallized (BuTPP)_2_MnBr_4_ crystals emit more strongly than the glassy counterpart, as shown in Figure 3c. A two-fold enhancement is cross-validated by the emission spectra (Figure 3d). This phenomenon aligns with our expectations that crystalline (BuTPP)_2_MnBr_4_ is more suitable for light emissions.

**Figure 4:**
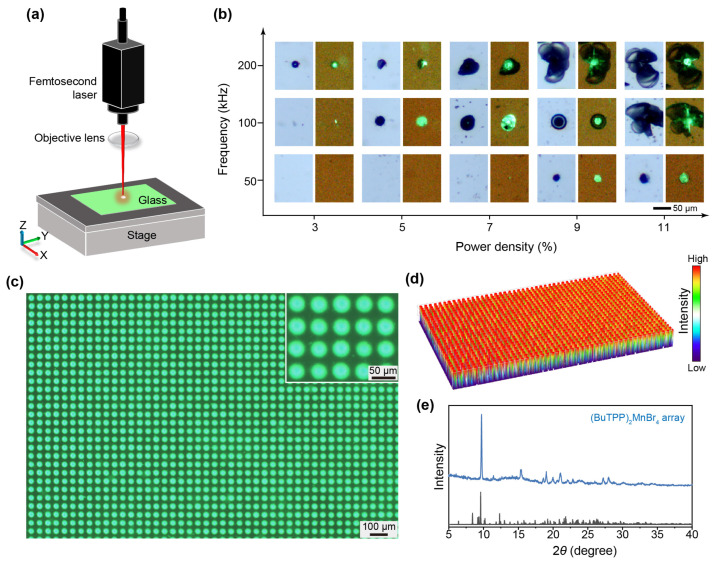
Fabrication and performance of the pixelated (BuTPP)_2_MnBr_4_ arrays through laser-induced in situ crystallization. (**a**) Schematic diagram of the preparation of pixelated (BuTPP)_2_MnBr_4_ arrays through a femtosecond-laser writing process. (**b**) Microscopic images of glass processed at different attenuation intensities and frequencies. (**c**) A fluorescent photograph of the pixelated (BuTPP)_2_MnBr_4_ arrays under 365 nm excitation. (**d**) Three-dimensional visualization of the pixel uniformity of the (BuTPP)_2_MnBr_4_ crystalline pixelated arrays. (**e**) The powder XRD pattern of (BuTPP)_2_MnBr_4_ crystalline pixelated arrays contains both crystalline and glassy phases.

Inspired by the thermally induced crystallization behavior, we consider that more precise in situ crystallization can be obtained by laser-writing (Figure 4a). Irradiation of the glass with a 1030 nm femtosecond laser produces highly localized energy deposition and a transient thermal field, thereby enabling controlled structural transformation within the irradiated region. To identify the processing window, we systematically varied the attenuation and repetition frequency while keeping the remaining conditions fixed (Figure 4b and Table S2). At low levels of both laser power and frequency, the interaction between the laser and glass is limited in intensity, making it difficult to detect discernible effects through conventional observation methods. Neither the surface morphology nor the optical property of the glass exhibits recognizable changes. When the laser frequency is set to 50 kHz, and the laser power is reduced to 7%, the interaction between the laser and the glass reaches an observable threshold. At this point, the effective effects of the laser on the glass can be clearly observed. The processed regions displayed regular morphological features and markedly enhanced PL under UV excitation, indicating the onset of local crystallization [41]. As laser frequency and power further increase, the energy density injected by the laser continues to rise, gradually expanding the interaction zone between the laser and the glass. Simultaneously, excessive energy input could exceed the glass’s tolerance threshold, causing significant structural damage in the glass matrix, accompanied by the formation and propagation of microcracks.

Based on the above optimization, the pixelated arrays were fabricated at a laser frequency of 100 kHz, a power attenuation of 5%, and a dot pitch of 50 μm. To evaluate the quality of laser-induced in situ crystallization, the pixelated (BuTPP)_2_MnBr_4_ arrays were examined by a fluorescence microscope. Under UV excitation, the PL intensity of the (BuTPP)_2_MnBr_4_ arrays exhibits a significant increase compared to glass, indicating that we have enhanced the luminescent properties of the material through laser processing (Figure 4c). We measured the light field of the pixel arrays under 365 nm excitation at the microscope, as shown in Figure 4d. The arrays resolved a highly uniform intensity profile with a remarkable fidelity, demonstrating their high homogeneity. The powder XRD of the (BuTPP)_2_MnBr_4_ arrays shows distinct crystalline peaks corresponding to those of the single crystal, verifying that laser processing has indeed generated a crystalline phase (Figure 4e). Note that the laser-induced pattern can be extended to other complex configurations, such as line-square, checkered, and ring-shaped patterns, as shown in Figure S2. Similarly, the processing areas are brighter than those of the glass areas under 365 nm excitation.

**Figure 5:**
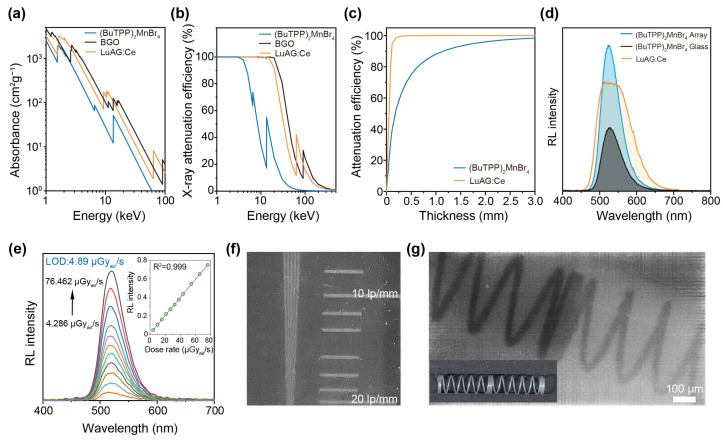
Performance of the scintillator based on pixelated (BuTPP)_2_MnBr_4_ arrays. (**a**) X-ray absorption coefficients plots of (BuTPP)_2_MnBr_4_ as a function of photon energies. Commercially available BGO and LuAG: Ce are used as references. (**b**) Plots of X-ray attenuation efficiency of (BuTPP)_2_MnBr_4_, BGO and LuAG:Ce as a function of photon energies. (**c**) Plots of calculated X-ray attenuation efficiency of (BuTPP)_2_MnBr_4_ and LuAG:Ce as a function of the thickness with 35 keV X-ray photons. (**d**) Comparison of the RL spectra for (BuTPP)_2_MnBr_4_ glass and crystalline pixelated arrays. LuAG:Ce is used as the reference (Tube voltage/current: 50 kV/70 μA). (**e**) The RL spectra of (BuTPP)_2_MnBr_4_ crystalline pixelated arrays as a function of X-ray dose rates. Inset: the linear relationship between X-ray dose rate and RL intensity. (**f**) X-ray imaging resolution measurement using crystalline pixelated arrays (Tube voltage/current: 50 kV/70 μA). (**g**) X-ray image of a spring. Inset: photograph of the spring under the natural light (Tube voltage/current: 50 kV/70 μA).

Given the excellent luminescence property, (BuTPP)_2_MnBr_4_ is anticipated to work as a high-quality scintillator. By integrating the pixelated (BuTPP)_2_MnBr_4_ arrays with a mature X-ray imaging system, we then tested their X-ray response. The X-ray absorption coefficient and attenuation efficiency of (BuTPP)_2_MnBr_4_ were carefully studied by comparing with commonly used scintillators (e.g., LuAG:Ce and BGO) on the market (Figure 5a–c). The X-ray attenuation efficiency of the (BuTPP)_2_MnBr_4_ glass is comparable to the state-of-the-art organic scintillators and a little inferior to the commercial inorganic scintillators (Figure 5b). In addition, the thickness-dependent X-ray absorption efficiency curve reveals that over 85% of the X-ray photons can be absorbed by using a ~1 mm thick (BuTPP)_2_MnBr_4_ (tube voltage: 35 kV) (Figure 5c).

According to the radioluminescence (RL) intensity in Figure 5d, the light yield of crystalline (BuTPP)_2_MnBr_4_ arrays is estimated to be 24,600 photons MeV^−1^, exceeding that of the glassy counterpart (9800 photons MeV^−1^) and slightly surpassing that of the standard scintillator LuAG:Ce with a light yield of 22,000 photons MeV^−1^ (Table S3). The RL intensity of the (BuTPP)_2_MnBr_4_ arrays at varying X-ray dose rates (tube voltage: 50 kV; tube current: 5–70 μA) was collected to evaluate the X-ray response limit (Figure 5e). It demonstrated excellent linearity within the X-ray radiation dose rate range of 4.3–76.4 μGy_air_ s^−1^ and a response limit of 4.89 µGy_air_ s^−1^, as shown in the inset of Figure 5e. Note that this value is smaller than that of the (BuTPP)_2_MnBr_4_ glass (Figure S3), and well below the typical threshold required for medical diagnostic imaging (<5.5 µGy_air_ s^−1^ as criterion). Meanwhile, the RL intensity maintained 90% of the initial value under X-ray radiation for over 1200 s, exhibiting high resistance to X-ray exposure (Figure S4). To assess the imaging performance, a lead line-pair card was used to determine the spatial resolution. As shown in Figure 5f, the spatial resolution of crystalline (BuTPP)_2_MnBr_4_ arrays is around 10 lp mm^−1^. We successfully acquired X-ray images of objects such as a metal spring (Figure 5g). The high-quality imaging demonstrates the practicality of crystalline (BuTPP)_2_MnBr_4_ arrays in practical X-ray imaging applications.

## 4. Conclusions

In summary, we developed a laser-induced in situ crystallization strategy to convert hybrid manganese(II) bromide glass into highly emissive pixelated arrays for X-ray imaging. (BuTPP)_2_MnBr_4_ exhibits favorable melt-processability, low-temperature glass formation, and thermally triggered recrystallization, enabling spatially selective crystal writing under femtosecond laser irradiation. Compared with the glassy state, the laser-crystallized (BuTPP)_2_MnBr_4_ arrays show enhanced photoluminescence, improved radioluminescence, and clear structural recovery of the crystalline phase. Benefiting from these features, the (BuTPP)_2_MnBr_4_ arrays deliver a high light yield of 24,600 photons MeV^−1^, an X-ray detection limit of 4.89 µGy_air_ s^−1^, and a spatial resolution of 10 lp mm^−1^, demonstrating strong promise for high-resolution scintillation imaging. This work provides a simple route for fabricating patterned hybrid metal-halide scintillators directly from glass precursors and highlights the potential of laser-written crystallization for integrated, customizable, and high-performance X-ray imaging devices based on low-temperature processable hybrid materials. The combination of processability, patternability, and imaging capability opens opportunities for high-performance scintillator platforms.

## Data Availability

The data presented in this study are available upon reasonable request.

## Supplementary Materials

The following supporting information can be downloaded at: https://www.mdpi.com/article/10.3390/s26082373/s1.
