# Supplementary material for: Laser-Induced In Situ Crystallization of Hybrid Manganese(II) Bromide Arrays for X-Ray Imaging
Source: Sensors (Basel). 2026 Apr 12;26(8):2373. doi: 10.3390/s26082373 (PMC13119750; doi:10.3390/s26082373)
Supplement: Supplementary file 1 [file sensors-26-02373-s001.zip › sensors-4233779-supplementary.pdf]

sensors

MDPI

Article

Laser-Induced In Situ Crystallization of Hybrid Manganese(II) Bromide Arrays for X-Ray Imaging

Zhaoran Lin [1], Guansheng Xing [1], Wei Wang [1,*] and Bing Chen [1,*]

[1] College of Electronic and Optical Engineering and College of Flexible Electronics (Future Technology),
Nanjing University of Posts and Telecommunications, Nanjing 210023, China

* Correspondence: wangwei29@njupt.edu.cn (W.W.); bchen@njupt.edu.cn (B.C.)

I. Supplementary Methods

1. X-ray attenuation coefficient and attenuation efficiency. The X-ray attenuation coefficient data of (BuTPP)2MnBr4, BGO and LuAG:Ce as a function of X-ray photon energy can be obtained by the XCOM database of the National Institute of Standards and Technology (NIST) according to the Equation (1)[1]:

$$AE(\%) = \left(1 - e^{-c(E)\rho d}\right) \times 100\% \tag{1}$$

where $c(E)$ denotes the total attenuation coefficient and ρ denotes the density (g/cm[3]) and d denotes the thickness (cm).

2. Light yield. The absolute photon number (P_{measured}) emitted by the sample under X-ray irradiation was collected through an integrating sphere and an Ocean Optics Spectrometer, coupled with a miniX2 X-ray tube (Amptek Inc.) as the excitation source. The photon measured was normalized to 100% X-ray attenuation using Equation (2)[1]:

$$P_{normalized} = \frac{P_{measured}}{AE(d)} \tag{2}$$

where AE(d) denotes the attenuation efficiency (%) of sample at its thickness. The light yield (LY) can be calculated by Equation (3):

$$LY(Sample) = LY_{LuAG:Ce} \times \frac{P_{normalized}(Sample)}{P_{normalized}(LuAG:Ce)} \tag{3}$$

The references are commercial LuAG:Ce, purchased from Shanghai Shuojie Crystal Materials Co., LTD.

3. Limit of Detection. The dose rates of the X-ray tube were carefully calibrated using the X-ray ion chamber dose meter, and different X-ray dose rates were achieved by adjusting the operating voltage or current of the X-ray source. The limit of detection was determined at a dose rate when the RL was three times the background noise.

4. X-ray imaging. A homemade X-ray imaging system comprises a portable X-ray source, a flat-panel scintillator, a reflector, and a complementary metal-oxide-semiconductor (CMOS) camera to record the X-ray images.

II. Supplementary Tables

Table S1. Single-crystal X-ray diffraction data of $(BuTPP)_2MnBr_4$

**Compound**	**$(BuTPP)_2MnBr_4$**
Empirical formula	$C_{44}H_{48}Br_4MnP_2$
Formula weight	1059.41
Temperature/K	293.0
Crystal system	monoclinic
Space group	$P2_1/n$
a/Å	10.8592
b/Å	21.7967
c/Å	19.6633
α/°	90
β/°	96.7430
γ/°	90
Volume/Å^3	4622.01
Z	4
P_{calc} g/cm^3	1.522
μ/mm^{-1}	3.846
F(000)	2132.0
Radiation	MoKα (λ = 0.71073)
2θ range for data collection/°	4.094 to 55.004
Index ranges	−14 ≤ h ≤ 14, −26 ≤ k ≤ 28, −25 ≤ l ≤ 24
Reflections collected	44228
Independent reflections	10611 [R_{int} = 0.0483, R_{sigma} = 0.0450]
Goodness-of-fit on F^2	1.017

Table S2. The laser-writing parameters for *in-situ* crystallization

**Power, W**	**Attenuation, %**	**Pulse count**	**Frequency, kHz**	**PSO**	**Stepping value**	**Pulse width, ps**	**Jump Speed, mm/s**
150	3	1	50	0	100	0.3	1
150	5	1	50	0	100	0.3	1
150	7	1	50	0	100	0.3	1
150	9	1	50	0	100	0.3	1
150	11	1	50	0	100	0.3	1
150	3	1	100	0	100	0.3	1
150	5	1	100	0	100	0.3	1
150	7	1	100	0	100	0.3	1
150	9	1	100	0	100	0.3	1
150	11	1	100	0	100	0.3	1
150	3	1	200	0	100	0.3	1
150	5	1	200	0	100	0.3	1
150	7	1	200	0	100	0.3	1
150	9	1	200	0	100	0.3	1
150	11	1	200	0	100	0.3	1

Table S3. A comparison of $(BuTPP)_2MnBr_4$ arrays with typical scintillators in terms of form and light yields.

Materials	Form	Light yield (photons/MeV)	Ref
LuAG:Ce	Crystal	22,000	Shanghai Shuojie Crystal Materials Co., LTD
BGO	Crystal	10,000	Shanghai Shuojie Crystal Materials Co., LTD
$(C_{25}H_{22}P)_2MnBr_4$	Crystal	78,937	[2]
$(TPT)_2MnBr_4$	Glass	20,060	[3]
(Hex-3,4-2F)$MnBr_4$	Glass	9,400	[4]
$(HTPP)_2MnBr_4$	Glass	8,600	[5]
$Cs_2AgInCl_6:Mn^{2+}$	Film	16,807	[6]
$Cs_2ZnBr_4:Mn^{2+}$	Film	15,600	[7]
$(BuTPP)_2MnBr_4$	Crystallized arrays	24,600	**This work**

II. Supplementary Figures

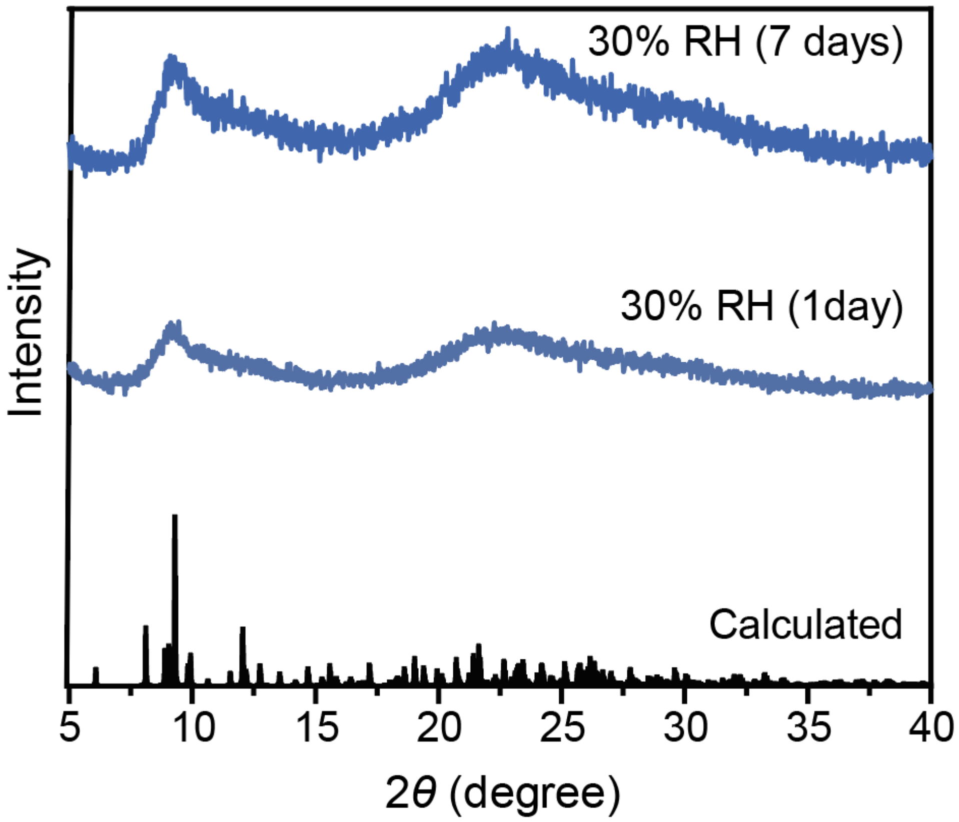

Figure S1. Powder XRD patterns of $(BuTPP)_2MnBr_4$ glass stored at 20 °C and a relative humidity of 30% for 1 and 7 days, respectively.

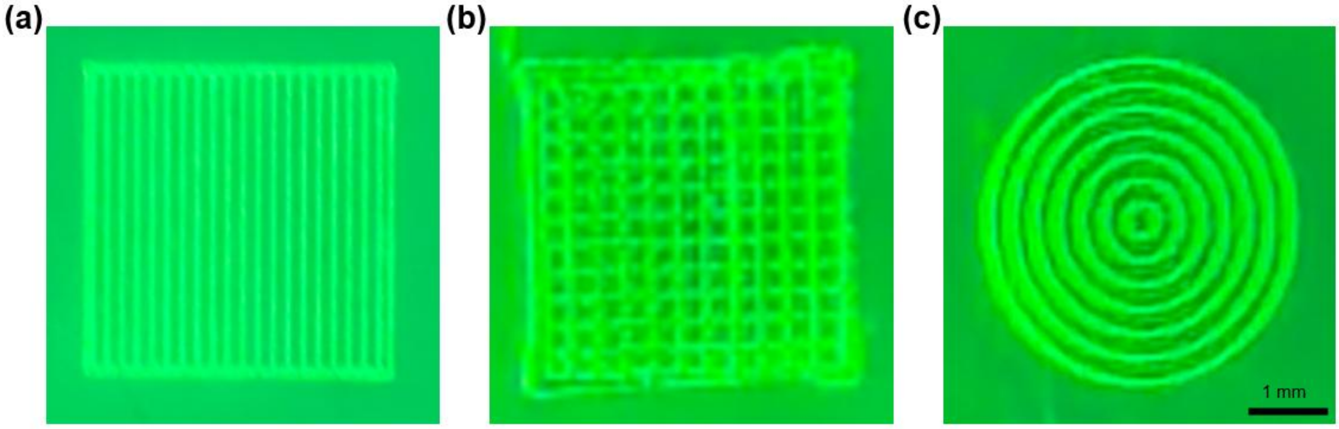

Figure S2. (a-c) The line-square, checkered, and ring-shaped patterns for $(BuTPP)_2MnBr_4$ glass after laser processing under 365 nm excitation, respectively.

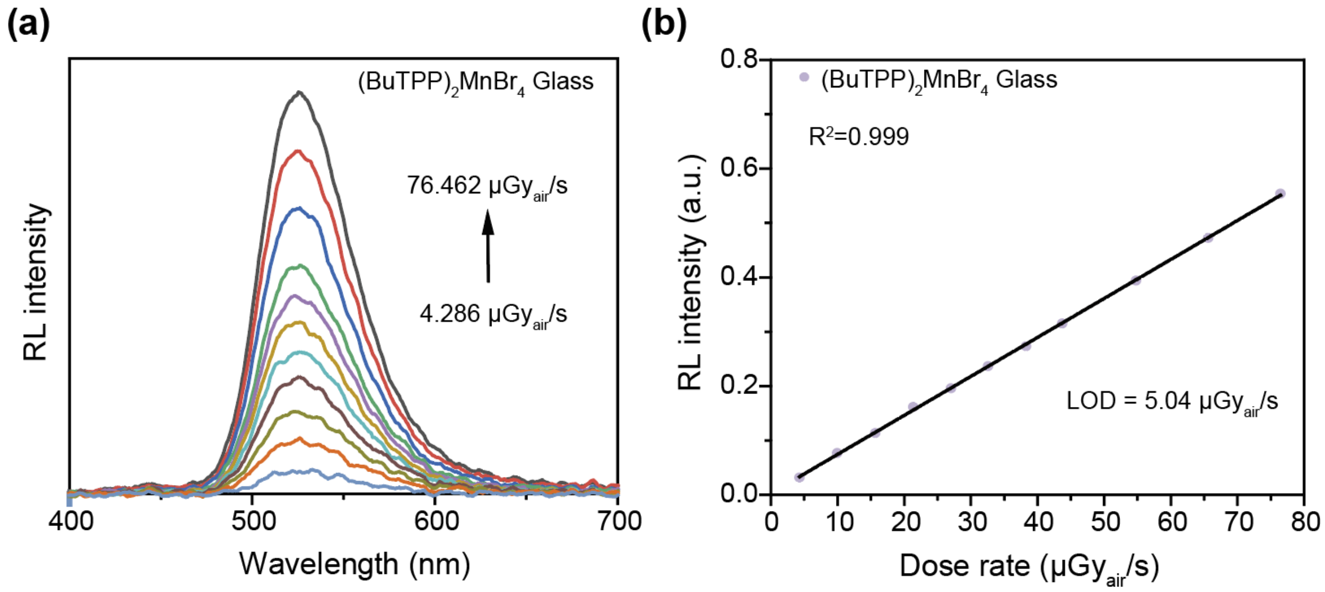

Figure S3. (a) The RL spectra of $(BuTPP)_2MnBr_4$ glass as a function of X-ray dose rates. (b) The linear relationship between X-ray dose rate and RL intensity.

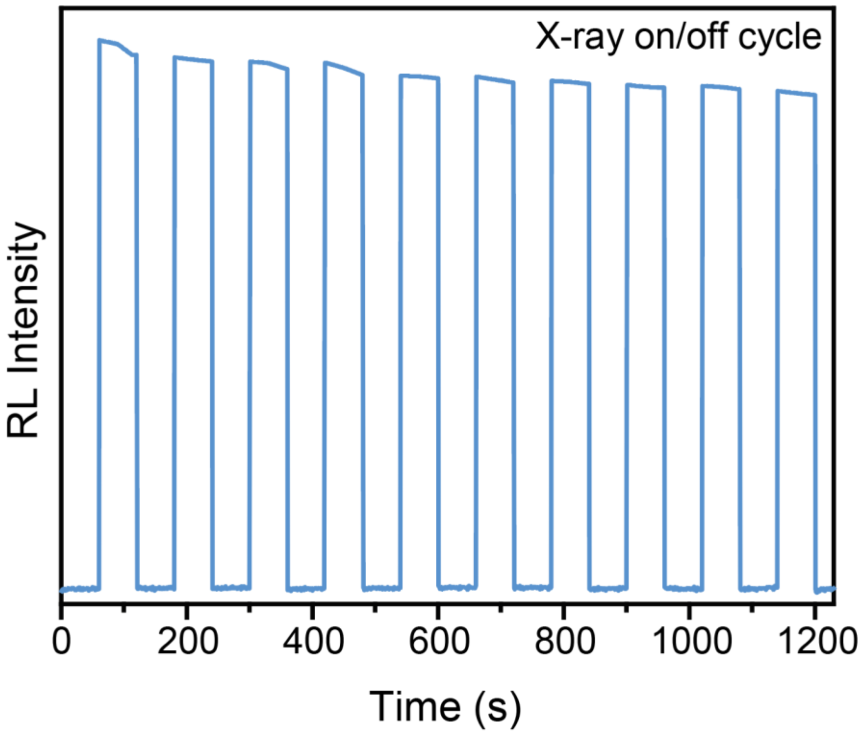

Figure S4. The radiation stability of crystalline $(BuTPP)_2MnBr_4$ arrays. The radiation stability was measured by recording the RL with an X-ray source (50 kV, 70 μA) on and off every 60 seconds.

References

1. Jiang, T.; Ma, W.; Zhang, H.; Tian, Y.; Lin, G.; Xiao, W.; Yu, X.; Qiu, J.; Xu, X.; Yang, Y.; Ju, D., Highly Efficient and Tunable Emission of Lead-Free Manganese Halides toward White Light-Emitting Diode and X-Ray Scintillation Applications. *Adv. Funct. Mater.* **2021,** *31(14)*, 2009973.
2. Zhou, M.; Jiang, H.; Hou, T.; Hou, S.; Li, J.; Chen, X.; Di, C.; Xiao, J.; Li, H.; Ju, D., Inch-size and thickness-adjustable hybrid manganese halide single-crystalline films for high resolution X-ray imaging. *Chem. Eng. J.* **2024,** *490*, 151823.
3. Jiang, J.; Mao, X.; Zheng, X.; Ye, X.; Li, C.; Li, H.; Ge, C.; Chen, J.; Wang, S.; Zhang, L.; Cui, S.; Lin, Q.; Guo, Q.; Han, Q.; Zhang, Y.; Tao, X.; Liu, Y., Reconfigurable Glass Scintillation Screen for Conformal X-Ray Imaging of Shape-Changeable Objects. *Laser Photonics Rev.* **2025,** *19(11)*, 2500266.
4. Luo, J.-B.; Wei, J.-H.; He, Z.-L.; Chen, J.-H.; Peng, Q.-P.; Zhang, Z.-Z.; Kuang, D.-B., Bisphosphonium cation based metal halide glass scintillators with tunable melting points. *Chem. Sci.* **2024,** *15(39)*, 16338–16346.
5. Luo, J. B.; Wei, J. H.; Zhang, Z. Z.; He, Z. L.; Kuang, D. B., A melt‐quenched luminescent glass of an organic‐inorganic manganese halide as a large‐area scintillator for radiation detection. *Angew. Chem.* **2023,** *135(7)*, e202216504.
6. Xing, G.; Cui, E.; Yuan, X.; Wang, B.; Chen, J.; Zhao, Y.; Tang, J.; Liu, J., Charge compensation via tetravalent doping for high-efficiency Mn^{2+}-activated inorganic double perovskites toward high-resolution X-Ray imaging. *Inorg. Chem. Front.* **2025,** *12(24)*, 8741–8749.
7. Su, B.; Han, K.; Xia, Z., Mn^{2+}-doped Cs_2ZnBr_4 scintillator for X-ray imaging. *J. Mater. Chem. C.* **2023,** *11(24)*, 8052–8061.
